# A new genus and species of clingfish from the Rangitāhua Kermadec Islands of New Zealand (Teleostei, Gobiesocidae)

**DOI:** 10.3897/zookeys.786.28539

**Published:** 2018-09-25

**Authors:** Kevin W. Conway, Andrew L. Stewart, Adam P. Summers

**Affiliations:** 1 Department of Wildlife and Fisheries Sciences and Biodiversity Research and Teaching Collections, Texas A&M University, College Station, TX 77843, USA; 2 Research Associate, Ichthyology, Australian Museum Research Institute, 1 William Street, Sydney, NSW 2010, Australia; 3 Museum of New Zealand Te Papa Tongarewa, 169 Tory Street, Wellington, New Zealand; 4 Friday Harbor Laboratories, University of Washington, Friday Harbor, WA 98250, USA; 5 Burke Museum of Natural History and Culture, University of Washington, Seattle, WA 98105, USA

**Keywords:** Acanthomorpha, Aspasminae, Diademichthyinae, Diplocrepinae, taxonomy

## Abstract

*Flexorincus*, new genus and species, is described from 15 specimens (14.0–27.2 mm SL) collected from shallow (0–9 meters) intertidal and sub-tidal waters of the Rangitāhua Kermadec Islands, New Zealand. The new taxon is distinguished from all other members of the Gobiesocidae by a combination of characters, including a heterodont dentition comprising both conical and distinct incisiviform teeth that are laterally compressed with a strongly recurved cusp, an oval-shaped opening between premaxillae, a double adhesive disc with a well-developed articulation between basipterygia and ventral postcleithra, and many reductions in the cephalic lateral line canal system. The new taxon is tentatively placed within the subfamily Diplocrepinae but shares a number of characteristics of the oral jaws and the adhesive disc skeleton with certain members of the Aspasminae and Diademichthyinae.

## Introduction

“*The discovery of this and several other new genera in recent years makes it necessary to reconsider the characterization and relationships of various subfamilies within the Gobiesocidae*” Briggs (1993: 197)

The family Gobiesocidae contains over 170 species and 50 genera of predominately small-bodied marine fishes found in coastal areas of the Atlantic and Indo-Pacific oceans (Briggs 1955; Conway et al. 2015), from the intertidal zone down to ~500 meters (Hastings and Conway 2017). Seven species also are known to inhabit freshwater streams in the Neotropics (Briggs and Miller 1960; Conway et al. 2017a). Commonly referred to as clingfishes, members of this family generally exhibit a well-developed ventral adhesive disc (formed by elements of the paired fins and paired-fin girdles; Guitel 1888), with which they can attach to smooth or even heavily structured substrates with great tenacity (Wainwright et al. 2013; Ditsche et al. 2014).

Clingfishes are considered archetypal crypto-benthic fishes (Brandl et al. 2018) and it is not surprising that new species continue to be discovered and described on an almost annual basis. Since 2010, this includes 19 new species (Fricke et al. 2010, 2015, 2016; Allen and Erdman 2012; Moore et al. 2012; Sparks and Gruber 2012; Conway et al. 2014, 2017b, c, 2018; Fricke 2014; Craig et al. 2015; Shinohara and Katayama 2015; Bilecenoğlu et al. 2017; Fricke and Wirtz 2017; Hastings and Conway 2017; Fujiwara and Motomura 2018; Fujiwara et al. 2018), three of which were also considered to represent new genera at the time of description (Fricke 2014; Fricke et al. 2016; Conway et al. 2017b).

Specimens of a reportedly new species of clingfish have been known from the remote Rangitāhua Kermadec Islands (here after Kermadec Islands) of New Zealand since at least 1980s (Francis et al. 1987; Francis 1993) and have been referred to in recent literature as *Aspasmogaster* sp. (Stewart 2015; Trnski et al. 2015) and by the common name “Kermadec clingfish” (Stewart 2015). These specimens differ markedly in a number of characters from the currently recognized species of *Aspasmogaster*, a genus reported to date only from temperate Australia (Hutchins 1984, 2008), and from members of other Australasian and Indo-Pacific genera of the Gobiesocidae. The purpose of the present paper is to provide a formal description for the “Kermadec clingfish”, which represents a new genus and species of the Gobiesocidae.

## Materials and methods

Specimens used in this study were obtained from the following museum collections:

**ANSP** Academy of Natural Sciences of Drexel University, Philadelphia

**AMS** Australian Museum, Sydney

**AIM** Auckland War Memorial Museum

**NMNZ** Museum of New Zealand Te Papa Tongarewa, Wellington

**ROM** Royal Ontario Museum, Toronto

**NMST-P** National Museum of Nature and Science, Tsukuba

**SAIAB** South African Institute of Aquatic Biodiversity, Grahamstown

**TCWC** Biodiversity Research and Teaching Collections, Texas A&M University, College Station

**USNM** National Museum of Natural History, Washington D.C.

**WAM**Western Australian Museum, Perth.

Head and body measurements reported follow Conway et al. (2014) and are expressed as percent of standard length (SL) or head length (HL). Adhesive disc papillae terminology follows Briggs (1955) and Hutchins (2008). Cephalic lateral line pore terminology follows Shiogaki and Dotsu (1983), except that we also use numbers to refer to individual pores following Conway et al. (2017b), with pores numbered along a particular canal from anterior to posterior or dorsal to ventral (lachrymal canal only). General osteological terminology follows that of Springer and Fraser (1976), except that we use the term anguloarticular instead of articular, anterior ceratohyal instead of ceratohyal, autopalatine instead of palatine, epicentral instead of epipleural (following Gemballa and Britz 1998), pharyngobranchial instead of infrapharyngobranchial, posterior ceratohyal instead of epihyal, and retroarticular instead of angular.

Select specimens were cleared and double stained (C&S) for bone and cartilage investigation using the protocol of Taylor and Van Dyke (1985). Select specimens were reversibly stained using cyanine blue following Saruwatari et al. (1997) to aid examination of adhesive disc papillae and cephalic lateral line canal pores. Specimens or parts thereof were observed and photographed using a ZEISS SteREO Discovery V20 stereomicroscope equipped with a ZEISS Axiocam MRc5 digital camera. Digital images were typically stacked using ZEISS Axiovision software. Computed tomography (CT) scans of select specimens were also obtained at the Karel F. Liem BioImaging Center (Friday Harbor Laboratories, University of Washington) using a Bruker (Billerica, MA) SkyScan 1173 scanner with a 1 mm aluminium filter at 60 kV and 110 μA on a 2240 × 2240 pixel CCD at a resolution of 8.8 μm. Specimens were scanned simultaneously in a 50ml plastic Falcon tube (Corning, NY), in which they were wrapped with cheesecloth moistened with ethanol (70%) to prevent movement during scanning. The resulting CT data were visualised, segmented, and rendered in Horos (www.horosproject.org) and Amira (FEI). The premaxilla and dentary from the right side were removed from select specimens and prepared for scanning electron microscopy (SEM) following the protocol outlined in Conway et al. (2015). Coated specimens were examined using a Tescan Vega3 SB scanning electron microscope. All digital images were processed using Adobe Photoshop and Adobe Illustrator.

## Systematics

### Flexor gen. n.

Taxon classificationAnimaliaGobiesociformesGobiesocidae

http://zoobank.org/23E32F56-CAF3-4B3D-938A-0BF6A590C070

#### Diagnosis.

A genus of the Gobiesocidae differing from all other genera by a combination of characters, including: head and anteriormost part of body similar in width; a relatively elongate body with a small, double adhesive disc located beneath anteriormost part of body; an oval-shaped gap between premaxillae formed by a semicircular indentation along medial edge of premaxilla; premaxilla with a single row of teeth, comprising 2–3 peg-like, conical teeth anteriorly at, and adjacent to, symphysis and 10–12 strongly laterally compressed, incisiviform teeth with strongly recurved cusp, along outer margin of bone; lower jaw with a single row of 14–16 small, conical teeth with sharply pointed and slightly recurved tip; posterior tip of basipterygium expanded and articulating with anteromedial edge of ventral postcleithrum via a shallow concave facet; mandibular portion of preoperculo-mandibular lateral line canal absent; lachrymal canal with two pores; upper and lower lip simple, uniform in thickness along jaw margin.

#### Etymology.

New Latin, anatomical term for muscles, from the Latin *flexus*, past participle of *flectere*, to bend. In reference to the great flexibility of clingfishes, many of which have the ability to bend the body so that the tail end comes to lie close to the head. Masculine.

#### Type species.

*Flexorincus*, new species

### Flexor incus sp. n.

Taxon classificationAnimaliaGobiesociformesGobiesocidae

http://zoobank.org/CFA9314B-78DB-4B46-9F7D-64BCD829E86D

Figs 1 , 2 , 3 , 4 , 5 , 6 , 7 , 8 , 9 , 10

Aspasmogaster sp.: Stewart 2015: 1539, 1544; Trnski et al. 2015: 473, 476, Table 1.

#### Holotype.

**NMNZ P.060717**, 20.8 mm SL; New Zealand, Kermadec Islands, Raoul Island, Fishing Rock Landing (29°15'03.0"S, 177°54'12.0"W), 0–1 meters depth, 18 May 2011, M. Francis.

#### Paratypes.

All Kermadec Islands. **AIM MA655142**, 1 (C&S), 20.0 mm SL; Raoul Island, North Meyer Islet, northwest side of island (29°14'40.4"S, 177°52'41.3"W), 0–1.3 meters depth, 13 May 2011. – **AIM MA655401**, 2, 18.0–24.0 mm SL; Raoul Island, Fishing Rock Landing (29°15'02.7"S, 177°54'11.7"W), 0–1 meters depth, 18 May 2011. – **AIM MA655316**, 1 (CT scan; https://doi.org/10.17602/M2/M56344), 23.0 mm SL; **AMS I.45807-001**, 1, 19.7 mm SL; **NMNZ P.049965**, 1, 24.3 mm SL; **NMNZ P.049966**, 1, 27.2 mm SL; Raoul Island, Herald Islets, west side of North Chanter Island (29°15'06.0"S, 177°51'21.0"W), 1–12 meters depth, 16 May 2011, A. Ballance. – **AMS I.45823-010**, 1, 19.1 mm SL; same as holotype. – **NMNZ P.017760**, 1, 14.0 mm SL; Raoul Island, rockpool on Fishing Rock Landing (29°15'S, 177°54'W) 17 Aug 1985. – **NMNZ P.041114**, 1, 22.4 mm SL; Raoul Island, Meyer Islet (29°14'48.0"S, 177°52'51.0"W), 0–1.5 meters depth, 7 November 2004. – **NMNZ P.024500**, 1, not measured; Fishing Rock, Raoul Island (29°10'S, 177°54'W), November 1980 – **NMNZ P.025315**, 1, 19.4 mm SL; Raoul Island (29°14'55.0"S, 177°58'22.6"W), 1975. – **NMNZ P.028570**, 1, 27.2 mm SL; Raoul Island, Meyer Islet, Boat Harbour (29°14'54.0"S, 177°52'12.0"W), 0–3 meters depth, 03 June 1992. – **NMNZ P.029570**, 1, 27.1 mm SL; Meyer Islet, Boat Harbour (29°14'54.0"S, 177°52'12.0"W), 03 June 1992. – **NMNZ P.050069**, 1, 21.1 mm SL; Raoul Island, Fishing Rock Landing (29°15'04.6"S, 177°54'12.9"W), 1 meter depth, 14 September 2011. – **NMNZ P.057561**, 1, 16.9 mm SL; Raoul Island (29°14'55.2"S, 177°58'22.8"W), 1975.

#### Diagnosis.

See generic diagnosis.

#### Description.

**Figure 1.**
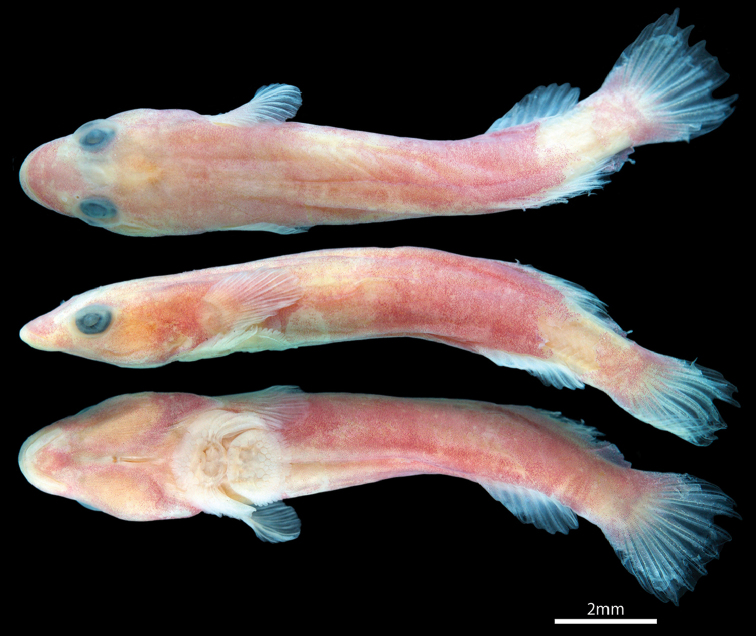
*Flexorincus*, NMNZ P.060717, holotype, 20.8 mm SL; New Zealand, Kermadec Islands, Raoul Island.

**Figure 2.**
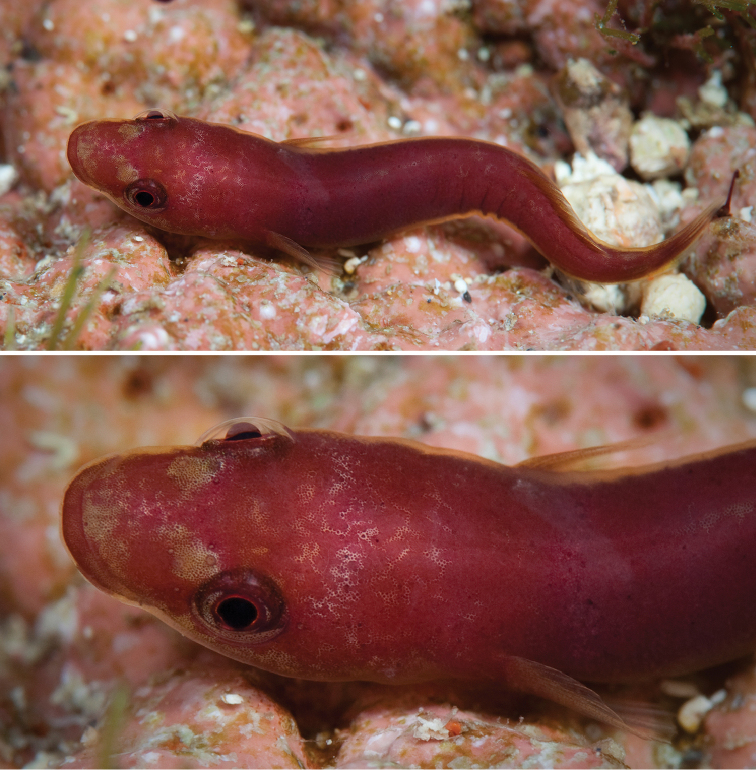
*Flexorincus*, Te konui Point, Raoul Island, Kermadec Islands, 28 meters depth, photographed by R. Robinson (www.depth.co.nz) during the 2011 Kermadec Islands Biodiscovery Expedition, a project led by the Auckland Museum. Specimen not retained.

**Figure 3.**
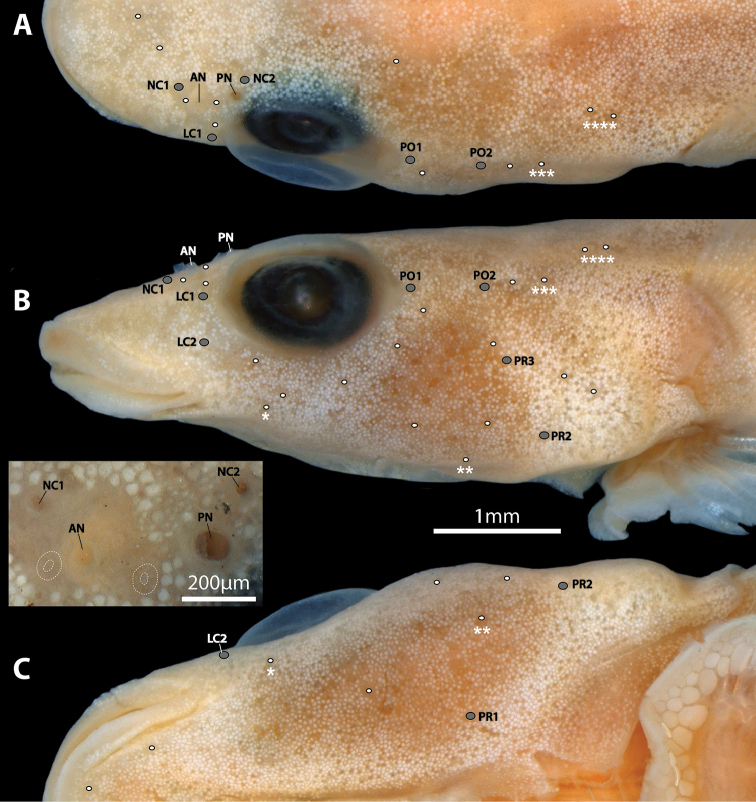
Head of *Flexorincus* (NMNZ P.025315, paratype, 19.4 mm SL) in dorsal (**A**), lateral (**B**), and ventral (**C**) view highlighting position of cephalic lateral line canal pores (grey circles) and superficial neuromasts (white circles) on the left side of the head. Close-up view of nostrils shown in inset image between **B** and **C** Superficial neuromasts highlighted by white dotted line in inset image. Single scale bar shared between **A–C**. Skin on ventral surface of head damaged on right side of specimen. Superficial neuromasts on surface of body not highlighted. Asterisks (*, **, ***, ****) label individual superficial neuromasts that are visible in different views. Abbreviations: AN, anterior nostril; LC1-2, lachrymal canal pores 1–2; NC1–2, nasal canal pores 1–2; PN, posterior nostril; PO1–2, postorbital canal pores 1–2; PR1–3, preopercular canal pores 1–3.

General body shape as in Figures 1, 2. Morphometric characters listed in Table 1. Head relatively small (less than one third of body length), slightly dorso-ventrally compressed. Body moderately elongate, circular in cross-section anteriorly, becoming increasingly laterally compressed posteriorly. Widest point of body immediately behind head; as wide as widest point of head. Body width tapering gradually posteriorly. Body depth relatively uniform anterior to dorsal and anal fins; shallowest along caudal peduncle. Eye large, positioned on dorsolateral surface of head; orbit barely visible in ventral view. Centre of eye closer to tip of snout than to posterior margin of operculum. Snout of moderate length, broad, anterior margin rounded (Figs 1–3). Anterior nostril a small tubular opening (Figure 3). Posterior nostril surrounded by a low, fleshy rim; situated along anterodorsal margin of orbit (Figure 3). Gill membranes free from isthmus.

**Table 1.** Select morphometric characters obtained from the holotype and six paratypes of *Flexorincus*. Ranges include value obtained for holotype.

	Holotype	Range	Mean	St. Dev.
Standard Length (SL)	20.8	16.9–27.2	–	–
**In % of SL**				
Head length (HL)	31.7	29.4–34.5	31.9	1.9
Body depth	14.9	10.0–14.9	13.6	1.9
Predorsal length	75.5	73.3–82.2	76.3	2.9
Preanal length	72.6	72.6–77.7	74.2	1.7
Preanus length	63.0	60.7–63.0	61.7	0.9
Anus to disc	15.9	14.1–18.6	16.8	1.5
Anus to anal fin	8.9	8.0–9.7	8.6	0.8
Caudal peduncle length	10.1	8.0–10.1	8.9	0.9
Caudal peduncle depth	11.1	8.2–11.1	9.4	0.9
Disc length	18.8	17.8–26.0	20.2	3
Disc width	16.3	16.3–20.1	17.7	1.3
**In % of HL**				
Head depth at orbit	34.8	25.9–34.8	29.1	3.4
Head width at orbit	47.0	42.6–51.9	47.6	3.1
Head width at widest point	60.6	52.9–67.3	58.3	5.4
Interorbital width	21.7	19.5–23.1	21.4	1.5
Snout length	30.3	28.1–36.5	32.2	2.7
Eye diameter	21.2	17.2–22.2	20.1	1.7

Mouth terminal, small; posterior tip of upper jaw reaching imaginary vertical line through anterior nostril when mouth closed. Upper and lower lip narrow; upper lip uniform in thickness along length of jaw; lower lip thicker along lateral margins of lower jaw, narrower at jaw symphysis (Figure 3). Upper jaw slightly wider and longer than lower jaw, creating a narrow gap between teeth of upper and lower jaws when jaws closed (Figure 5). Premaxilla with a single row of teeth, comprising 2–3 peg-like, conical teeth anteriorly at and adjacent to symphysis, and 10–12 strongly laterally compressed incisiviform teeth, each with a single strongly recurved cusp, along outer margin of bone (Figure 7A–C). Dentary with a single row of 14–16 small, conical teeth with sharply pointed and slightly recurved tip (Figure 7D). Pharyngeal jaws comprising patch of 4–6 small conical teeth with slightly recurved tips on pharyngobranchial toothplate 3 and row of 3–5 small conical teeth with slightly recurved tips along ceratobranchial 5 (Figure 8D). 5–6 small triangular gill rakers located along anterior and posterior edge of ceratobranchials 2–3 and anterior edge of ceratobranchial 4; one or two tiny gill rakers located along anterior edge of ceratobranchial 1 (Figure 8D). Paired rows of gill filaments (holobranch) on gill arches I-III (three gill filaments of Briggs 1955). Basihyal an elongate rod, widest posteriorly at point of articulation with dorsal hypohyals; anterior edge tipped with cartilage (Figure 8C). Six brachiostegal rays (Figure 8A); anteriormost ray separate from hyoid bar; second ray articulating medially with hyoid bar along anterior ceratohyal; posterior rays articulating with hyoid bar laterally, including two along posteriormost part of anterior ceratohyal, one straddling junction between anterior and posterior ceratohyals, and posteriormost along anteriormost part of posterior ceratohyal (Figure 8B). Anteriormost branchiostegal ray shorter than posterior rays. Three posteriormost branchiostegal rays similar in width and length, approximately twice as long and thick as anteriormost ray. Intervening rays intermediate in width and length (Figure 8B).

Cephalic lateral-line system with 2 pores in nasal canal; 2 pores in postorbital canal; 2 pores in lachrymal canal; 3 pores in preopercular canal (Figure 3). Mandibular canal absent. Canal pores minute; typically flush with surface of skin and difficult to locate. Supraorbital canals (including nasal canal plus anteriormost region of postorbital canal of Shiogaki and Dotsu 1983) connected across midline via epiphyseal commissure (Figure 6A). Superficial neuromasts on surface of head not arranged in obvious rows (Figure 3); each surrounded by a shallow groove.

**Figure 4.**
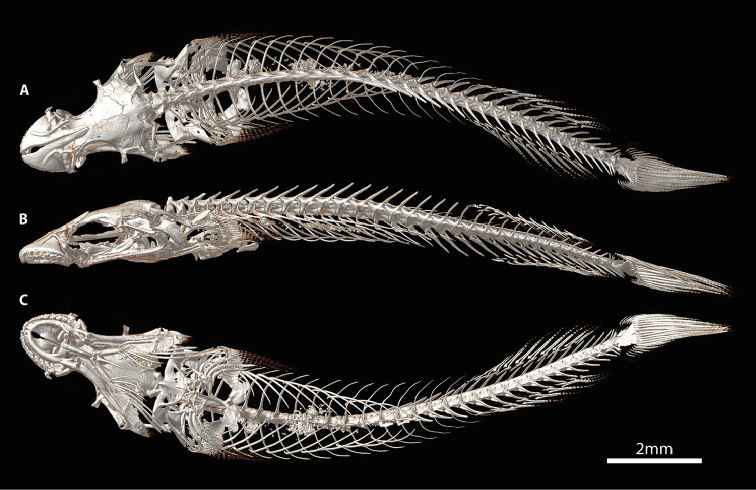
CT scanned skeleton of *Flexorincus*, AIM MA655316, paratype, 23.0 mm SL. **A** Dorsal view. **B** Lateral view (left side) **C** Ventral view.

Dorsal-fin rays 9 or 10. Anal-fin rays 8 or 9 (first in serial or supernumerary association with anteriormost proximal-middle radial). Principal caudal-fin rays 5+5, dorsal procurrent rays 6 or 7, ventral procurrent rays 6. Pectoral-fin rays 24 or 25; uppermost ray a tiny splint-like element comprised of a single hemitrichium. Pelvic-fin rays I, 4. All fins rays, excluding anteriormost 4–5 procurrent caudal-fin rays, unbranched and segmented; anteriormost 4–5 procurrent caudal-fin rays unsegmented, azygous elements. Caudal fin marginally truncate, tips of principal caudal-fin rays extended beyond fin margin. Caudal-fin skeleton comprised of upper and lower hypural plates; epural triangular, with broad cartilaginous dorsal margin; parhypural absent, parahypural cartilage roughly triangular (Figure 10). Neural and hemal spine of PU2 bifurcated in single CT scanned specimen (Figure 4); singular in C&S specimen (Figure 10). Dorsal-fin origin situated slightly anterior to imaginary vertical line through anal-fin origin (Figs 1, 4). First dorsal-fin pterygiophore inserted between neural spines of vertebrae 17/18. First anal-fin pterygiophore inserted between hemal spines of vertebrae 18/19 or 19/20. Total number of vertebrae 33, consisting of 13 or 14 abdominal vertebrae and 19 or 20 caudal vertebrae (Figure 4). Ribs 12, associated with vertebrae 3–14. Epicentrals 20, associated with vertebrae 2–21.

**Figure 5.**
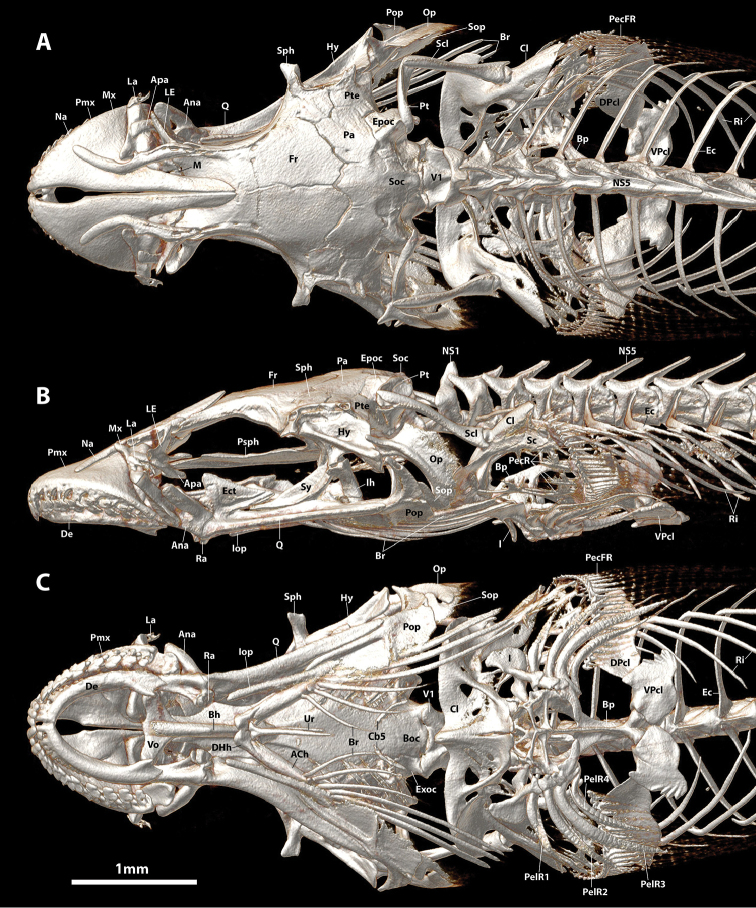
CT scanned anterior skeleton of *Flexorincus*, AIM MA655316, paratype, 23.0 mm SL. **A** Dorsal view **B** Lateral view (left side) **C** Ventral view. Abbreviations: ACh, anterior ceratohyal; Ana, anguloarticular; Apa, autopalatine; Boc, basioccipital; Bp, basipterygium; Br, branchiostegal rays; Cb5, ceratobranchial 5; Cl, cleithrum; DHh, dorsal hypohyal; DPcl, dorsal postcleithrum; Ec, epicentral; Ect, ectopterygoid; Epoc, epiotic; Exoc, exoccipital; Fr, frontal; Hy, hyomandibular; I, pelvic-fin spine; Iop, interopercle; La, lachrymal; LE, lateral ethmoid; M, mesethmoid; Na, nasal; NS1, 5, neural spine of vertebral centrum 1, 5; Op, opercle; Pa, parietal; PecR, pectoral radial; PecFR, pectoral-fin ray; PelFR1-4, pelvic-fin ray 1–4; Pop, preopercle; Pro, prootic; Psph, parasphenoid; Pt, posttemporal; Pte, pterotic; Q, quadrate; Ra, retroarticular; Ri, rib; Sc, scapula; Scl, supracleithrum; Soc, supraoccipital; Sop, subopercle; Sph, sphenotic; Ur, urohyal; V1, vertebral centrum 1; Vo, vomer; VPcl, ventral postcleithrum.

**Figure 6.**
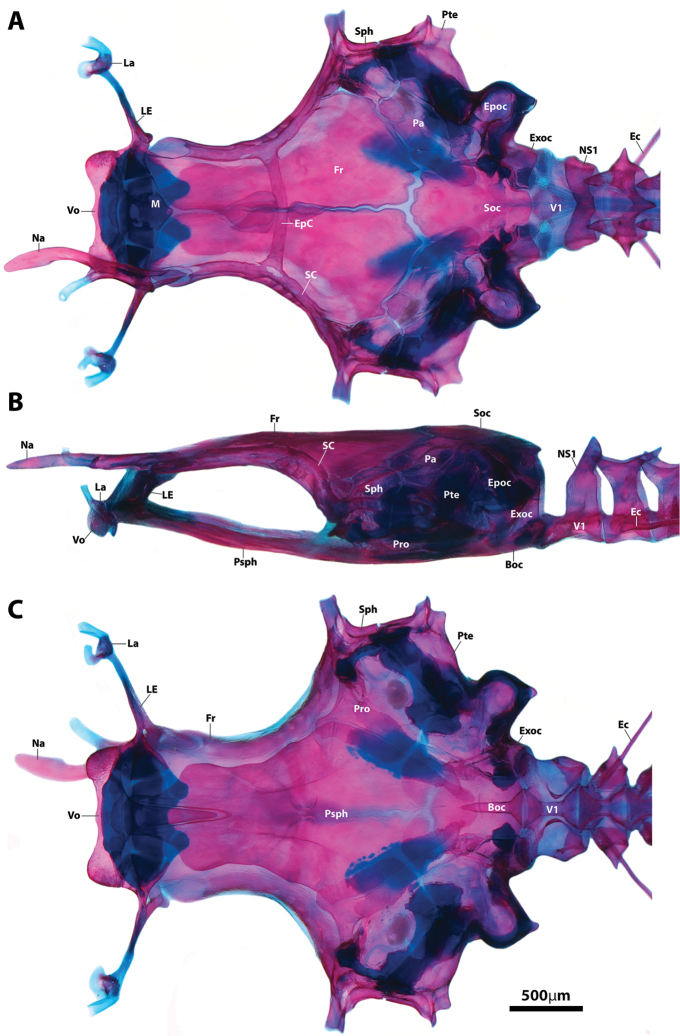
Neurocranium of *Flexorincus*, AIM MA655142, paratype, 20.0 mm SL. **A** Dorsal view **B** Lateral view (left side) **C** Ventral view. Abbreviations: Boc, basioccipital; Ec, epicentral; EpC, epiphyseal commissure of supraorbital canal; Epoc, epiotic; Exoc, exoccipital; Fr, frontal; La, lachrymal; LE, lateral ethmoid; M, mesethmoid; Na, nasal; NS1, neural spine of vertebral centrum 1; Pa, parietal; Pro, prootic; Psph, parasphenoid; Pte, pterotic; SC, supraorbital canal; Soc, supraoccipital; Sph, sphenotic; V1, vertebral centrum 1; Vo, vomer.

**Figure 7.**
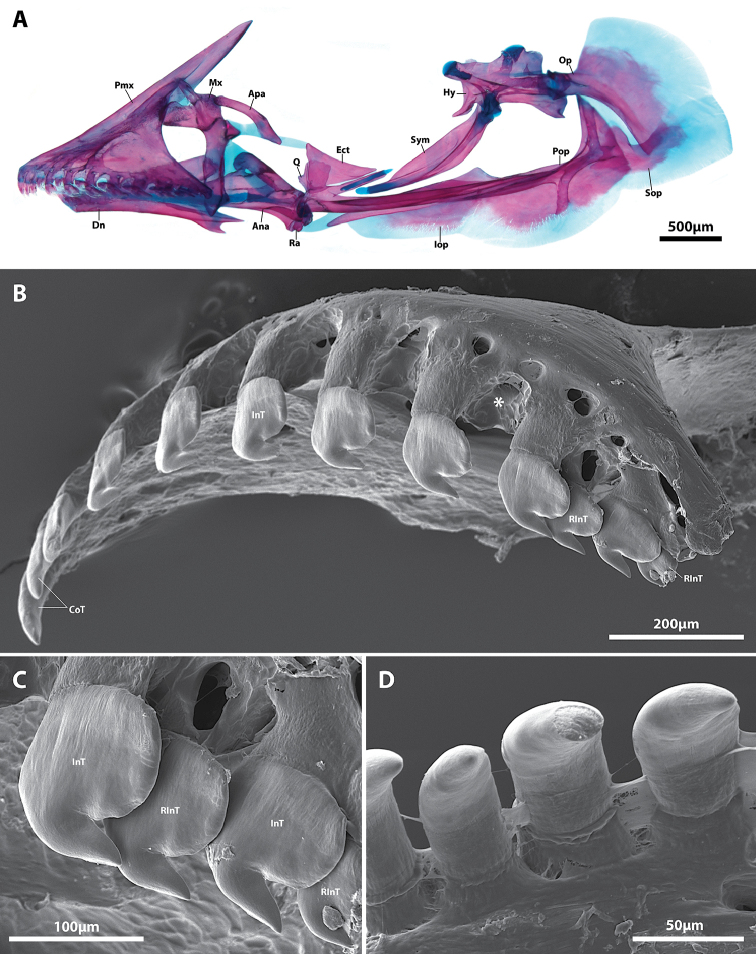
*Flexorincus*, AIM MA655142, paratype, 20.0 mm SL. **A** Hyopalatine arch and opercular series, right side in lateral view (image reversed) **B** Scanning electron micrograph of premaxilla, right side in oblique lateral view (image reversed). Asterisk (*) highlights location of replacement tooth crypt **C** Close up of posteriormost incisiviform teeth of premaxilla shown in **A D** Scanning electron micrograph of conical teeth located close to middle of dentary, right side in medial view (image reversed). Abbreviations: Ana, anguloarticular; Apa, autopalatine; CoT, conical tooth; Dn, dentary; Ect, ectopterygoid; Hy, hyomandibular; InT, incisiviform tooth; Iop, interopercle; Mx, maxilla; Op, opercle; Pop, preopercle; Q, quadrate; Ra, retroarticular; RInT, replacement incisiviform tooth; Sop, subopercle; Sym, symplectic.

**Figure 8.**
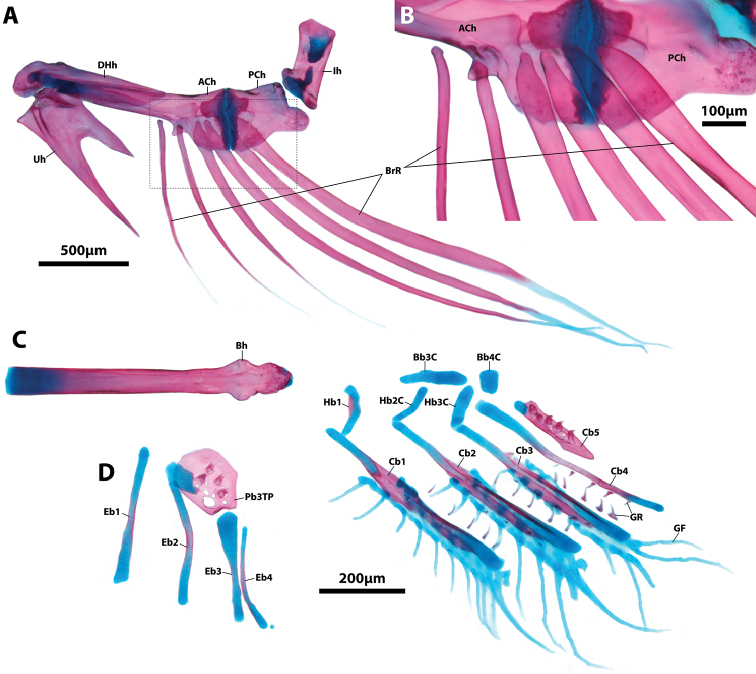
Hyoid bar (**A–B**) and gill arches (**C–D**) of *Flexorincus*, AIM MA655142, paratype, 20.0 mm SL. **A** Hyoid bar, right side in lateral view (image reversed) **B** Close up of box in **A** showing articulating between heads of branchiostegal rays and hyoid bar **C** Lower gill arches in dorsal view, paired elements of right side omitted **D** Upper gill arches of left side in ventral view. A single scale bar shared between **C** and **D**. Abbreviations: ACh, anterior ceratohyal; Bb3-4C, basibranchial 3 or 4 cartilages; Bh, basihyal; BrR, branchiostegal rays; Cb1-5, ceratobranchial 1-5; DHh, dorsal hypohyal; EB1-4, epibranchials 1-4; GF, gill filament; GR, gill raker; Hb1, hypobranchial 1; Hb2-3C, hypobranchial 2 or 3 cartilage; Pb3TP, pharyngobranchial 3 toothplate; Uh, urohyal.

**Figure 9.**
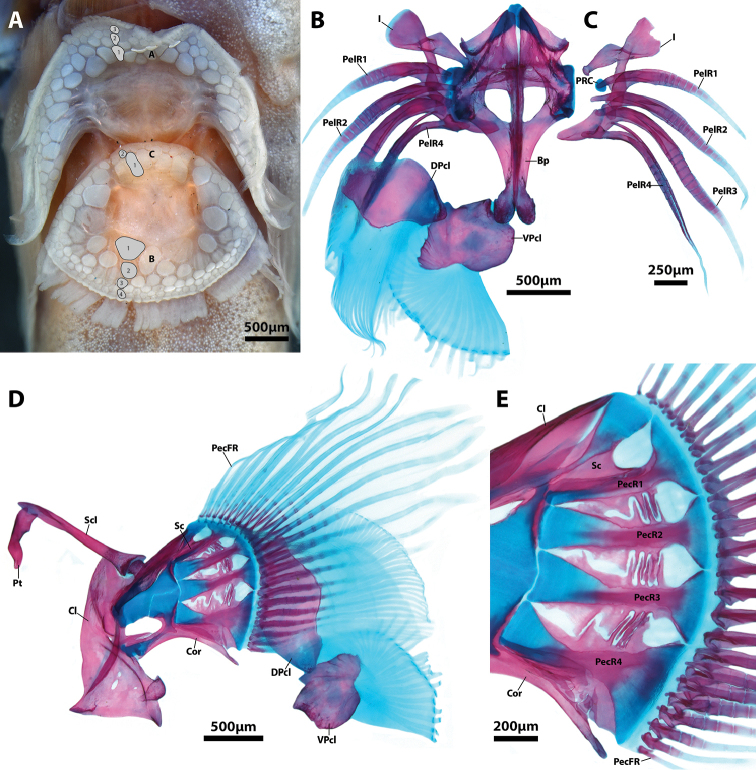
Surface features of the adhesive disc (**A**NMNZ P.025315, paratype, 19.4 mm SL) and internal supporting skeleton of the paired-fin girdles (**B–E**AIM MA655142, paratype, 20.0 mm SL) of *Flexorincus*. **A** Adhesive disc in ventral view (anterior to top of page); a single row of papillae highlighted in grey in each region (A–C) of the adhesive disc **B** Adhesive disc supporting skeleton, including elements of the pelvic and pectoral-fin girdle in dorsal view (anterior to top of page); postcleithra and pelvic-fin rays of the right side removed **C** Pelvic-fin rays of right side in dorsal view (anterior to top of page) **D** Pectoral-fin girdle of right side in medial view (anterior to left) **E** Close-up of elements of the pectoral-fin endoskeleton articulating with pectoral-fin rays of the right side in medial view (anterior to left). Abbreviations: A, disc region A; B, disc region B; Bp, basipterygium; C, disc region C; DPcL, dorsal postcleithrum; I, pelvic-fin spine; PecR1–4, pectoral radial 1–4; PecFR, pectoral-fin ray; PelR1–4, pelvic-fin rays 1–4 VPcL, ventral postcleithrum.

Adhesive disc small (18–26% of SL), double (Figure 9A); anterior margin weakly crenulated medially, becoming smooth at point corresponding to location of expanded tip of spinous ray; posterior margin bordered by a broad, thin and weakly crenulated skin flap. Skin of posterior flap delicate, easily damaged; supported internally by fimbrae of ventral postcleithrum. Disc region A with 3–4 transverse rows of papillae. Disc region B with 4–5 transverse rows of papillae. Disc region C with 2–3 rows of papillae. Papillae of disc region A decreasing in diameter gradually towards outer margin of disc. Papillae of disc region B and C decreasing in diameter towards outer margin of inner disc. Decrease in size of papillae of disc region C abrupt, with papillae of inner row 2–3 times larger than papillae of outer rows. Dorsal postcleithrum a well ossified shield-shaped bone with ~25 long, poorly ossified and distally bifurcated fimbrae (Figure 9B). Ventral postcleithrum well ossified, irregular in shape; approximately equal in size to dorsal postcleithrum (Figure 9B). ~20 long, poorly ossified and distally bifurcated fimbrae restricted to posterolateral margin of ventral postcleithrum; point of fimbrae bifurcation located distally on medial fimbrae, shifting gradually to a more proximal location on lateral fimbrae. A strong articulation between anteromedial edge of ventral postcleithrum and posterior tip of basipterygium (Figs 5B, 9B). Skin associated with last pelvic-fin ray attaching to base of pectoral fin opposite 5^th^–6^th^ lowermost pectoral-fin rays. Skin over base of ventral pectoral-fin rays smooth. Pectoral radials with well-developed bony struts along ventral (pectoral radial 1), dorsal (pectoral radial 4), or both ventral and dorsal margins (pectoral radials 2 and 3) that interdigitate with struts borne on element(s) directly above and/or below (Figure 9D, E).

**Figure 10.**
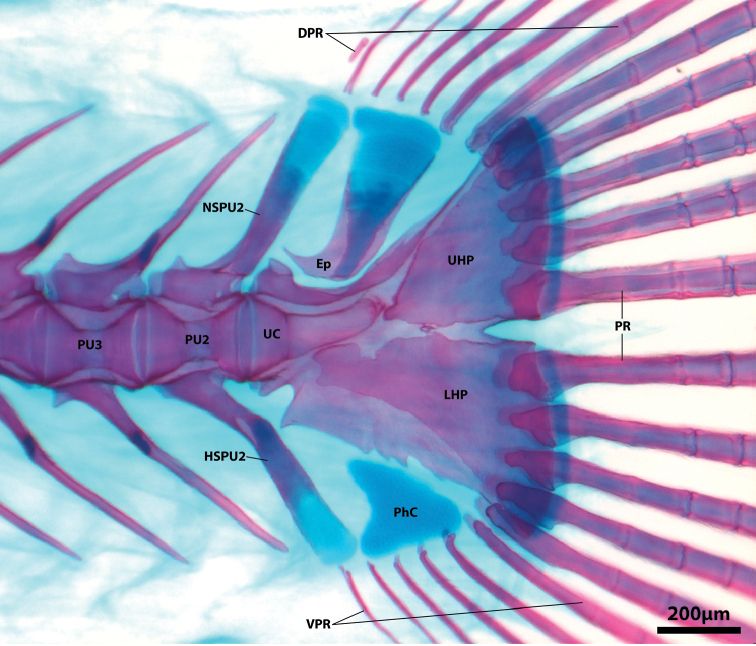
Caudal-fin skeleton (left side, lateral view) of *Flexorincus*, AIM MA655142, paratype, 20.0 mm SL. Abbreviations: DPR, dorsal procurrent caudal-fin rays; Ep, epural; HSPUS, hemal spine of preural centrum 2; LHP, lower hypural plate; NSPU2, neural spine of preural centrum 2; PhC, parhypural cartilage; PR, principal caudal-fin ray; PU2-3, preural centrum 2, 3; UC, ural centrum; UHP, upper hypural plate; VPR, ventral procurrent caudal-fin ray.

**Figure 11.**
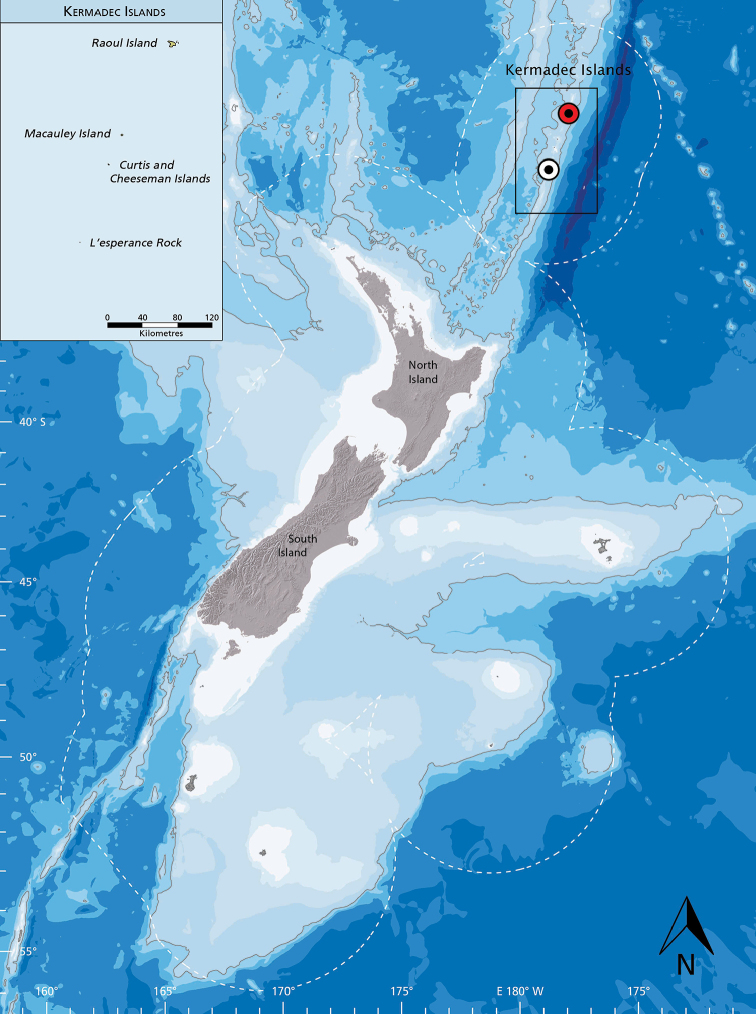
Distribution of *Flexorincus*. Type locality in red.

**Colouration.** In alcohol, head and body background colour typically uniformly pale cream to yellow (Figure 3). Holotype (Figure 1) has retained pinkish purple colour of live individuals and is an exception. Fins hyaline.

In life (Figure 2), body uniformly pinkish purple to grey, with diffuse, pale markings ranging from bars to irregular blotches. Head pinkish purple to grey, with diffuse, pale areas around nostrils and tip of snout. Iris red. Fins transparent.

**Distribution and habitat.** Known to date only from intertidal and subtidal waters of the Kermadec Islands (Figure 11), including Raoul Island (type locality) and L’Esperance Rock. The majority of available specimens were collected from rock pools and from shallower subtidal areas (down to 9 meters) over rock and coral rubble substrates using ichthyocides (Stewart 2015). However, a single specimen of the new species has been observed (and photographed) at 28 meters in depth (Figure 2).

**Etymology.***Incus* is the Latin word for anvil, in reference to the anvil-like outline of Raoul Island, the largest island in the Kermadec archipelago and type locality of the new species. A noun in apposition.

**Figure 12.**
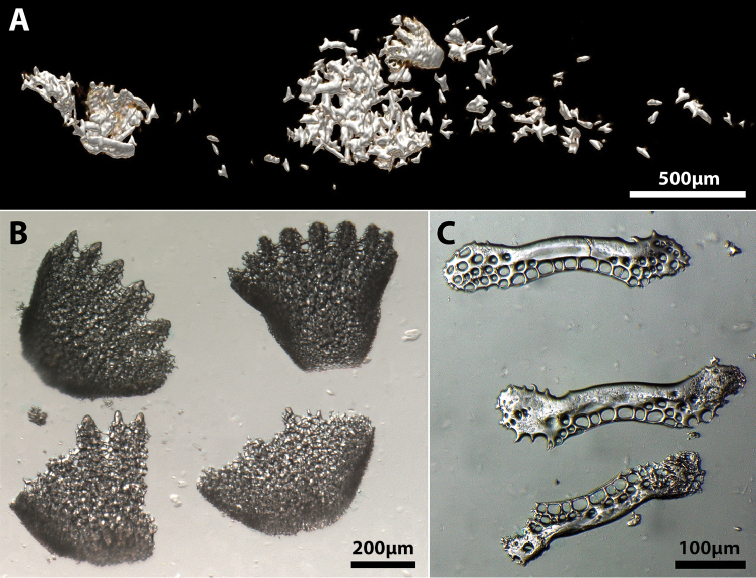
Gut content of *Flexorincus.***A** Hard gut content of AIM MA655316, paratype, 23.0 mm SL, as revealed by CT scanning. Isolated elements from the gut of AIM MA655142, paratype, 20.0 mm SL**B** Stereomic ossicles tentatively identified as elements from the terminal disc of an echinoid **C** Rod-like elements tentatively identified as ossicles from the body of a holothuroid.

**Gut content.** Hard and irregular shaped items ranging in size from 50–300 μm are scattered throughout the stomach of the CT scanned individual (Figure 12A). Smaller elements have smooth surfaces and could not be identified. Several of the larger elements appear to exhibit a porous (potentially stereomic) surface and are tentatively identified as echinoderm remains. Hard elements inside the stomach of the single C&S individual survived the clearing and staining process and could be dissected and photographed (Figure 12B, C). These elements are tentatively identified as either stereomic ossicles from the terminal disc of an echinoid (Figure 12B) or ossicles from the body of a holothuroid (Figure 12C) suggesting that the new species consumes echinoderms or parts of echinoderms.

## Discussion

The Kermadec Islands are a group of tiny remote islands almost 1,000 km from New Zealand, about half way between Tonga and Auckland, and lie between 29°15'S and 31°21'S. The Kermadec-Tonga arc is the longest submarine arc on the planet, running 2,500 km along the boundary of the Pacific and Australian Plates. It is a region of high geothermal activity with 83% of investigated volcanic centres venting (de Ronde et al. 2007). Directly to the east of the ridge lies the Kermadec Trench, the fourth deepest in the world which reaches to ca 10,000 meters deep at the deepest point. The Kermadec Islands constitute a series of four small emergent islands (Raoul, Macauley, Curtis, and Nugent) and two large rocks (L’Esperance and Havre). The largest of these islands is Raoul Island (2,040 ha, rising to 520 m asl) and the smallest L’Esperance Rock (4.8 ha, 70 m als) (Trnski and de Lange 2015). Havre Rock only just breaks the surface at low tide. The total land area of these islands and rocks is ca. 33 km^2^ (Mortimer and Campbell 2014). The cones have had a highly dynamic recent history with explosive emergence and collapse (Lloyd et al. 1996). In spite of geothermal activity and remoteness, the Kermadec Ridge and adjacent trench is inhabited by over 2,000 taxa (Duffy and Ahyong 2015) including 397 species of fishes (Te Papa unpublished records), of which nine are known to be endemic to the shelf (0–200 m depth). These include *Anarchiassupremus*, *Microbrotulapunicea*, *Lepidotriglarobinsi*, *Hypoplectrodes* sp. n., *Enneapterygiuskermadecensis*, *Eviotakermadecensis*, *Arnoglossus* sp. n., *Lophonectes* sp. n., and the new clingfish species described herein (Roberts et al. 2015; Duff and Ahyong 2015; Te Papa unpublished records).

**Figure 13.**
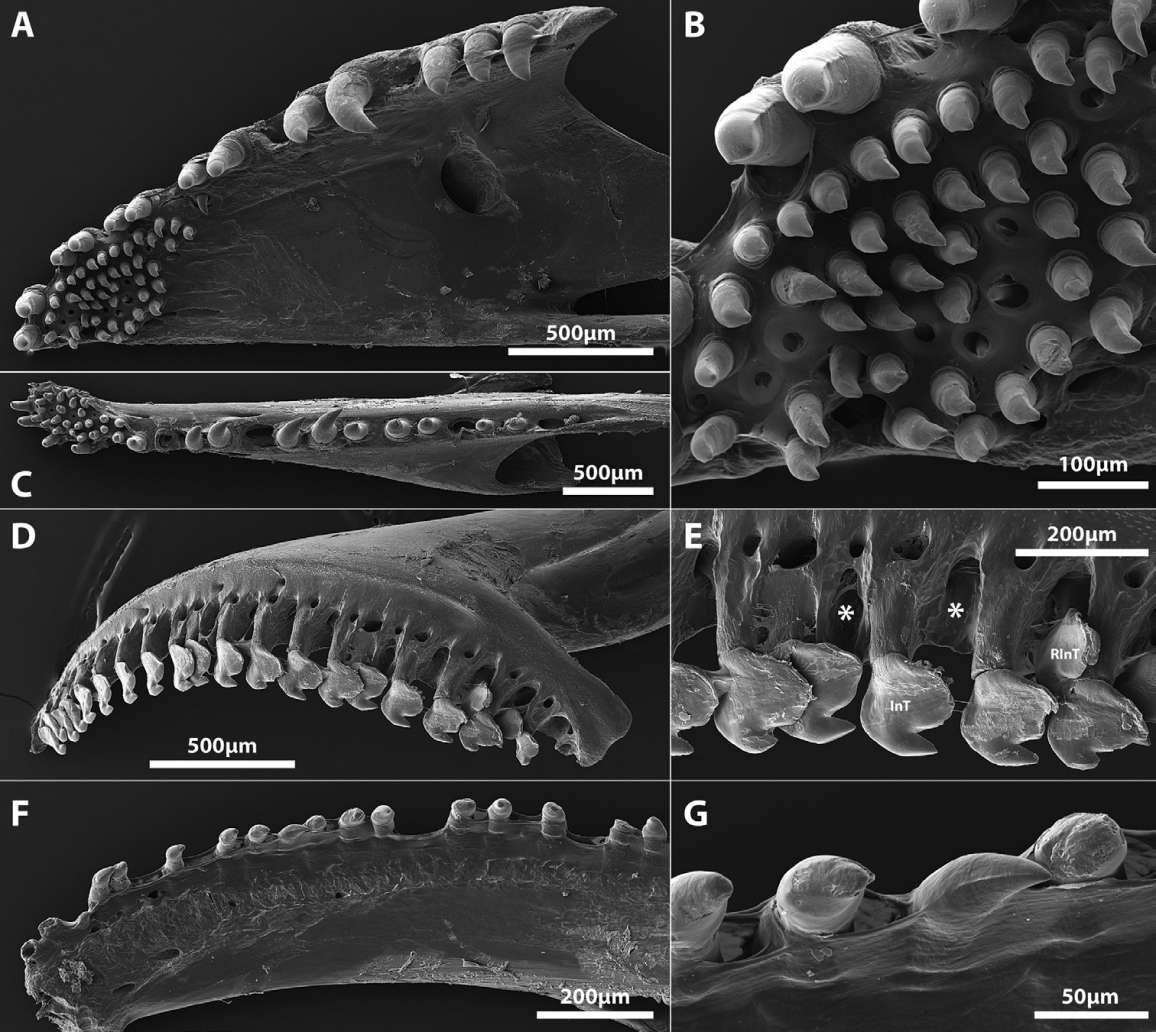
Scanning electron micrographs of the tooth-bearing oral jaw bones of *Aspasmogastercostata* (AMS I.19103-015, 32.0 mm SL) and *Lepadichthyscoccinotaenia* (SAIAB 49396, 31.0 mm SL). **A** Premaxilla of *A.costata*, right side in ventral view (image reversed) **B** Close up of lingual toothpatch on premaxilla of *A.costata* shown in **A C** Dentary of *A.costata*, right side in ventral view (image reversed) **D** Premaxilla of *Lepadichthyscoccinotaenia*, right side in lateral view (image reversed) **E** Close up of incisiviform teeth located on posterior part of premaxilla of *L.coccinotaenia* shown in **D** Asterisks (*) highlight locations of crypts associated with dislodged replacement teeth **F** Dentary of *Lepadichthyscoccinotaenia*, right side in medial view **G** Close up of conical teeth located along midregion of dentary of *L.coccinotaenia* shown in **F** Abbreviations: InT, incisiviform tooth; RInT, replacement incisiviform tooth.

**Figure 14.**
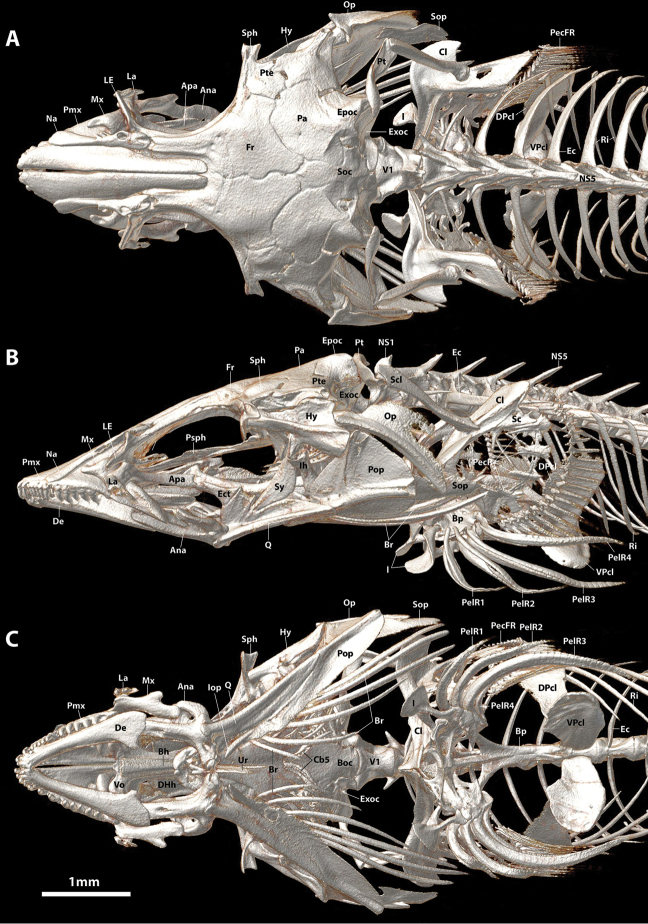
CT scanned anterior skeleton of *Aspasmogastercostata*, TCWC 17166.01, 30.0 mm SL. **A** Dorsal view **B** Lateral view (left side) **C** Ventral view. Abbreviations: ACh, anterior ceratohyal; Ana, anguloarticular; Apa, autopalatine; Boc, basioccipital; Bp, basipterygium; Br, branchiostegal rays; Cb5, ceratobranchial 5; Cl, cleithrum; DHh, dorsal hypohyal; DPcl, dorsal postcleithrum; Ec, epicentral; Ect, ectopterygoid; Epoc, epiotic; Exoc, exoccipital; Fr, frontal; Hy, hyomandibular; I, pelvic-fin spine; Iop, interopercle; La, lachrymal; LE, lateral ethmoid; Na, nasal; NS1, 5, neural spine of vertebral centrum 1, 5; Op, opercle; Pa, parietal; PecR, pectoral radial; PecFR, pectoral-fin ray; PelFR1-4, pelvic-fin ray 1–4; Pop, preopercle; Pro, prootic; Psph, parasphenoid; Pt, posttemporal; Pte, pterotic; Q, quadrate; Ra, retroarticular; Ri, rib; Sc, scapula; Scl, supracleithrum; Soc, supraoccipital; Sop, subopercle; Sph, sphenotic; Ur, urohyal; V1, vertebral centrum 1; Vo, vomer; VPcl, ventral postcleithrum.

Specimens of *Flexorincus* have been referred to previously as *Aspasmogaster* sp. (Stewart 2015; Trnski et al. 2015). This taxonomic assignment, considered “tentative” by Stewart (2015), was based on preliminary attempts to identify the new species by B. Hutchins in 1980s (A. Stewart pers. obs.). *Aspasmogaster* is currently represented by four species (viz. *A.costata*, *A.liorhynchus*, *A.occidentalis* and *A.tasmaniensis*) and restricted to coastal areas of temperate Australia (Hutchins 1984; Hutchins 2008). The new species differs most obviously from *Aspasmogaster* by features of the oral jaw dentition, including both the arrangement (teeth in both jaws arranged in a single row vs. teeth in both jaws arranged in a broad patch anteriorly, tapering to a single row posteriorly; Figure 13A, C) and the type of teeth present on the premaxilla (*Flexor* is a heterodont with both conical and incisiviform teeth on the premaxilla [Figure 7A, B] vs. premaxilla with conical teeth only [Figure 13A, B]). It can be further distinguished from *Aspasmogaster* by having an oval opening between the premaxillae formed by a characteristic indentation along the medial edge of each premaxilla (vs. medial edge of premaxilla straight, premaxillae abutting along entire medial edge or separated only by a narrow gap [Figure 14A]), simple lips, both of which are relatively thin and uniform in thickness along the length of the jaws (vs. lower lip expanded adjacent to the symphysis into a prominent fleshy fold in all four species of *Aspasmogaster*; upper lip also expanded and overlapping anterolateral margin of snout in *A.occidentalis*; Hutchins 1984), by features of the adhesive disc, including a lower number of transverse rows of papillae in all disc regions (3–4 rows in region A, 4–5 in region B and 2–3 in region C vs. 5–8 rows in region A, 6–9 in region B and 3–5 in region C; Briggs 1955; Hutchins 1984), a well-developed articulation between the posterior tip of the basipterygium and the anteromedial edge of the ventral postcleithrum (vs. basipterygium and ventral postcleithrum without contact; Figure 14C, see also Hutchins 1984: figure 5), and by features of the cephalic lateral line canals, including the absence (vs. presence) of the mandibular portion of the preoperculo-mandibular canal, and 2 (vs. 3) openings in the lachrymal canal.

Based on the characters listed in the key to the subfamilies of the Gobiesocidae (Briggs 1955: 10), *Flexor* would be considered a member of the Diplocrepinae, which in addition to *Aspasmogaster* is also hypothesised to include *Cochleoceps*, *Diplocrepis*, *Gastrocyathus*, *Gastrocymba*, *Gastroscypthus*, *Parvicrepis*, *Pherallodus* and *Propherallodus* (Briggs 1955; Shiogaki and Dotsu 1983; Hardy 1984; Fujiwara and Motomura 2018). The composition of this subfamily has been questioned previously by Briggs (1993) and we suspect that it is not monophyletic (see below). Regardless, *Flexor* can be distinguished from all of the aforementioned genera except *Pherallodus* and *Propherallodus* by features of the adhesive disc, including the absence (vs. presence) of papillae in region D and by having a well-developed articulation between the posterior tip of the basipterygium and the anteromedial edge of the ventral postcleithrum (vs. basipterygium and ventral postcleithrum without contact or with simple contact). It can be further distinguished from all but *Pherallodus* by the presence of strongly laterally compressed incisiviform teeth with a strongly recurved cusp, along the outer margin of the premaxilla (vs. simple peg-like conical teeth or strongly recurved conical teeth along the outer margin of the premaxilla), and from *Pherallodus* by the presence (vs. absence) of the preopercular portion of the preoperculo-mandibular canal, the lower jaw with conical teeth only (vs. lower jaw with both conical and incisiviform teeth), and by a complete field of papillae across the centre of region A and C of the adhesive disc (vs. papillae absent from centre of both region A and C) (Shiogaki and Dotsu 1983).

**Figure 15.**
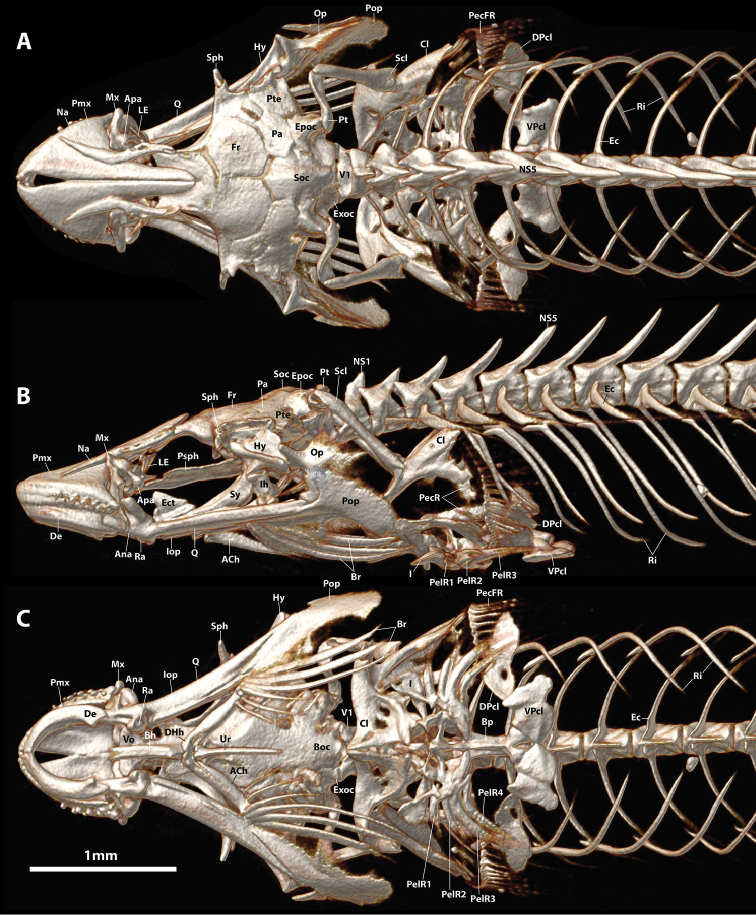
CT scanned anterior skeleton of *Pherallodusindicus*, NSMT-P 114703, 25.0 mm SL. **A** Dorsal view **B** Lateral view (left side) **C** Ventral view. Abbreviations: ACh, anterior ceratohyal; Ana, anguloarticular; Apa, autopalatine; Boc, basioccipital; Bp, basipterygium; Br, branchiostegal rays; Cl, cleithrum; DHh, dorsal hypohyal; DPcl, dorsal postcleithrum; Ec, epicentral; Ect, ectopterygoid; Epoc, epiotic; Exoc, exoccipital; Fr, frontal; Hy, hyomandibular; I, pelvic-fin spine; Iop, interopercle; LE, lateral ethmoid; Na, nasal; NS1, 5, neural spine of vertebral centrum 1, 5; Op, opercle; Pa, parietal; PecR, pectoral radial; PecFR, pectoral-fin ray; PelFR1-4, pelvic-fin ray 1–4; Pop, preopercle; Pro, prootic; Psph, parasphenoid; Pt, posttemporal; Pte, pterotic; Q, quadrate; Ra, retroarticular; Ri, rib; Scl, supracleithrum; Soc, supraoccipital; Sph, sphenotic; Ur, urohyal; V1, vertebral centrum 1; Vo, vomer; VPcl, ventral postcleithrum.

**Figure 16.**
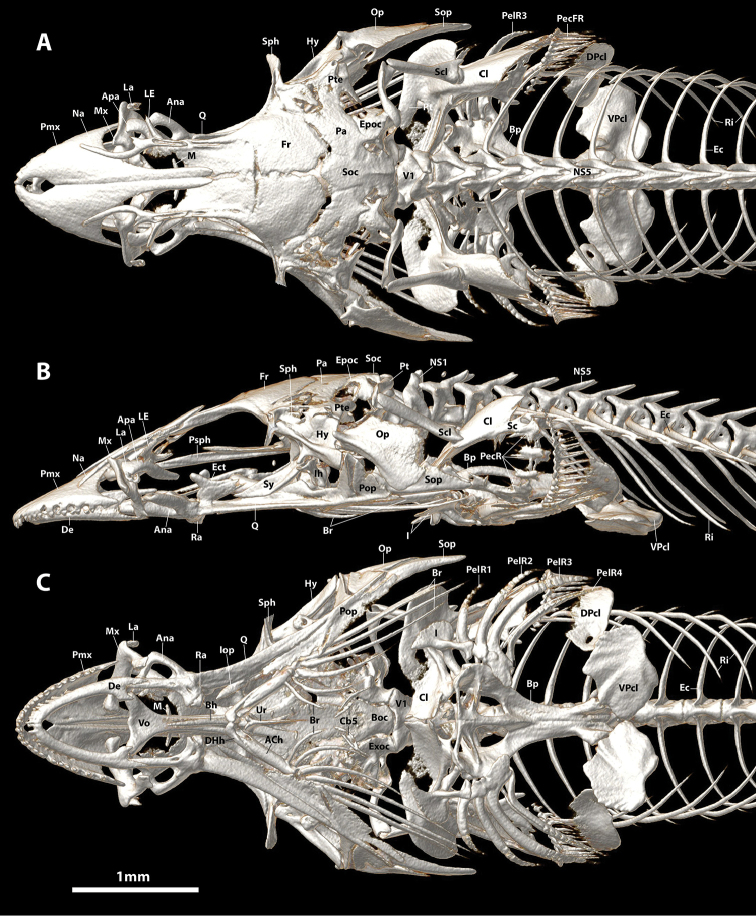
CT scanned anterior skeleton of *Aspasmichthysciconiae*, TCWC 16461.02, 26.0 mm SL. **A** Dorsal view **B** Lateral view (left side) **C** Ventral view. Abbreviations: ACh, anterior ceratohyal; Ana, anguloarticular; Apa, autopalatine; Boc, basioccipital; Bp, basipterygium; Br, branchiostegal rays; Cb5, ceratobranchial 5; Cl, cleithrum; DHh, dorsal hypohyal; DPcl, dorsal postcleithrum; Ec, epicentral; Ect, ectopterygoid; Epoc, epiotic; Exoc, exoccipital; Fr, frontal; Hy, hyomandibular; I, pelvic-fin spine; Iop, interopercle; La, lachrymal; LE, lateral ethmoid; M, mesethmoid; Na, nasal; NS1, 5, neural spine of vertebral centrum 1, 5; Op, opercle; Pa, parietal; PecR, pectoral radial; PecFR, pectoral-fin ray; PelFR1-4, pelvic-fin ray 1–4; Pop, preopercle; Pro, prootic; Psph, parasphenoid; Pt, posttemporal; Pte, pterotic; Q, quadrate; Ra, retroarticular; Ri, rib; Sc, scapula; Scl, supracleithrum; Soc, supraoccipital; Sop, subopercle; Sph, sphenotic; Ur, urohyal; V1, vertebral centrum 1; Vo, vomer; VPcl, ventral postcleithrum.

**Figure 17.**
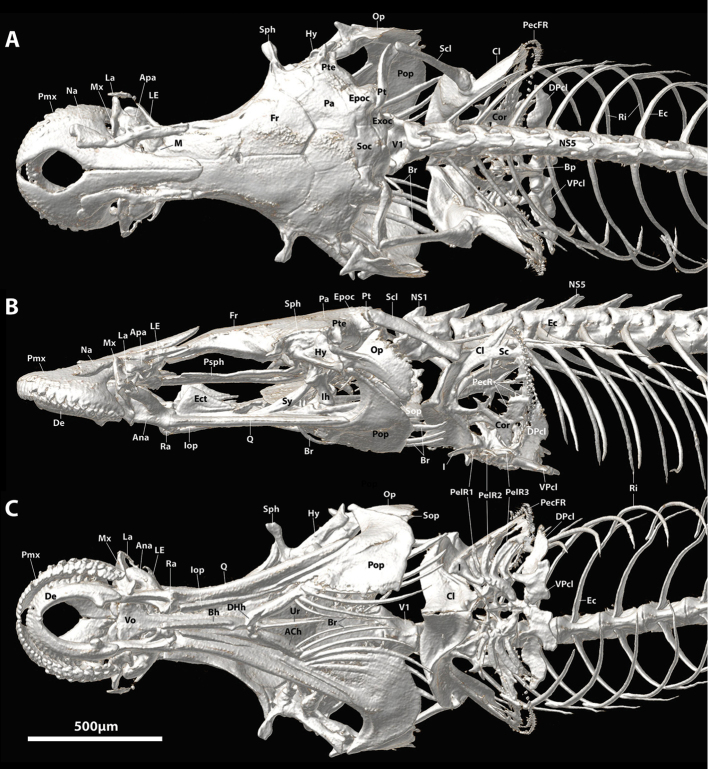
CT scanned anterior skeleton of *Lepadichthysbolini*, ROM 55185, 21.5 mm SL. **A** Dorsal view **B** Lateral view (left side) **C** Ventral view. Abbreviations: ACh, anterior ceratohyal; Ana, anguloarticular; Apa, autopalatine; Bp, basipterygium; Br, branchiostegal rays; Cb5, ceratobranchial 5; Cl, cleithrum; Cor, coracoid; DHh, dorsal hypohyal; DPcl, dorsal postcleithrum; Ec, epicentral; Ect, ectopterygoid; Epoc, epiotic; Exoc, exoccipital; Fr, frontal; Hy, hyomandibular; I, pelvic-fin spine; Iop, interopercle; La, lachrymal; LE, lateral ethmoid; M, mesethmoid; Na, nasal; NS1, 5, neural spine of vertebral centrum 1, 5; Op, opercle; Pa, parietal; PecR, pectoral radial; PecFR, pectoral-fin ray; PelFR1-4, pelvic-fin ray 1–4; Pop, preopercle; Pro, prootic; Psph, parasphenoid; Pt, posttemporal; Pte, pterotic; Q, quadrate; Ra, retroarticular; Ri, rib; Sc, scapula; Scl, supracleithrum; Soc, supraoccipital; Sop, subopercle; Sph, sphenotic; Ur, urohyal; V1, vertebral centrum 1; Vo, vomer; VPcl, ventral postcleithrum.

The characteristic type of incisiviform tooth present on the premaxilla of *Flexor* (Figure 7B, C) and *Pherallodus* is also present in some members of the Aspasminae (*Aspasmichthys* and *Pherallodichthys*) and the Diademichthyinae (*Diademichthys* and *Lepadichthys*) (Briggs 1955; Shiogaki and Dotsu 1983; Hayashi et al. 1986). This distinct type of incisiviform tooth appears to have been first described and illustrated by Briggs (1955: figure 71) for *Aspasma*, *Aspasmichthys, Diademichthys* and *Lepadichthys*. Briggs (1955) described these teeth as “highly compressed with reversed points [sic]” (pg. 137) or “broad with pointed reverse tips [sic]” (pg. 141). We have been unable to observe this characteristic type of incisiviform tooth in our C&S material of *Aspasmaminima* (NMST-P 114701) but our observations on the dentition of *Aspasmichthys*, *Diademichthys* and *Lepadichthys* are congruent with those of Briggs (1955). In *Pherallodichthysmeshimaensis, Aspasmichthysciconiae* (Figure 16B), and some members of *Lepadichthys* (e.g., *L.bolini*; Figure 17B) the incisiviform teeth are arranged in a single row along the outer margin of the premaxilla and, as in *Flexor*, are combined with a small number of peg-like conical teeth anteriorly (Shiogaki and Dotsu 1983; Hayashi et al. 1986). In other members of *Lepadichthys* (e.g., *L.frenatus* and *L.coccinotaenia*; Figure 13D, E) the entire upper jaw comprises only a single row of ca. 18–20 incisiviform teeth, though there is a clear gradation in the width of the incisiviform teeth and the distinctiveness of the tooth cusp along the length of the upper jaw, with those located more anteriorly being narrower with a less defined cusp than those located posteriorly (Figure 13D). In *Diademichthys*, incisiviform teeth are arranged in a single row along both the dentary and premaxilla (Briggs 1955; Hayashi et al. 1986, Figure 7H, 8H). In addition to this characteristic incisiviform tooth, *Aspasmichthys*, *Diademichthys*, *Lepadichthys*, *Pherallodichthys* and *Pherallodus* also share a complex articulation between the posterior tip of the basipterygium and the anteromedial edge of the ventral postcleithrum with *Flexor*. Additionally, and excluding *Pherallodichthys*, these taxa also share a characteristic oval opening between the premaxillae formed by a semicircular indentation along the medial edge of each premaxilla. This opening is relatively small and restricted to the anterior part of the premaxilla only in *Flexor* (Figure 5A, C), *Pherallodus* (Figure 15A, C), *Aspasmichthys* (Figure 16A, C) and *Diademichthys* (see Briggs 1955: fig. 81; Hayashi et al. 1986: fig. 7H) but is greatly enlarged in the species of *Lepadichthys* that we have examined (excluding *L.lineatus* in which the premaxillae are unmodified) and encompassing almost the anterior 1/3 of the upper jaw (Figure 17A). In some species of *Lepadichthys* (e.g., *L.frenatus*) the semicircular indentation extends anteriorly to the symphysis of the upper jaw, where the premaxillae are without contact (Briggs 1955). In this extreme condition, the majority of the anterior part of the upper jaw is roofed only by skin.

We consider the aforementioned characters of the oral jaws ([1] characteristic incisiviform teeth and [2] characteristic oval opening between the premaxillae formed by a semicircular indentation along the medial edge of each premaxilla) and the complex articulation between the posterior tip of the basipterygium and the anteromedial edge of the ventral postcleithrum as derived characteristics and putative evidence that the aforementioned members of the Diplocrepinae (*Flexor*, *Pherallodus*), Aspasminae (*Aspasmichthys* and *Pherallodichthys*), and Diademichthyinae (*Diademichthys* and *Lepadichthys*) are potentially more closely related to each other than they are to other members of these subfamilies that do not exhibit these characters. If correct, the relationships of *Flexor* would lie with Indo-Pacific taxa and not with the members of the endemic New Zealand gobiesocid fauna (Stewart 2015), as is the case for many other Kermadec endemic shore fishes, including *Enneapterygiuskermadecensis*, which is considered a member of either the *E.hemimelas*-group (Fricke 1994) or *E.pyramis*-group (Fricke 1997), both with members distributed broadly through the eastern Pacific (from Taiwan/Ryukyu Islands to Lord Howe Island), and *Eviotakermadecensis*, which is most similar and potentially closely related to species of *Eviota* from Japan (Hoese and Stewart 2012).

We started our paper with a quote from John C. “Jack” Briggs (1920–2018): “The discovery of this and several other new genera in recent years makes it necessary to reconsider the characterization and relationships of various subfamilies within the Gobiesocidae” Briggs (1993: 197). We agree wholeheartedly.

## Comparative material (C&S and/or CT scanned material only)

**Aspasminae – *Aspasma***: *A.minima* – USNM 270219, 1 (C&S), 40.2 mm SL; NSMT-P 114701, 2 (C&S), 35.0–51.0 mm SL. ***Aspasmichthys***: *A.ciconiae* – TCWC 16461.02, 1 (CT, https://doi.org/10.17602/M2/M30821), 26.0 mm SL. ***Pherallodichthys***: *P.meshimaensis* – NSMT-P 46753, 1 (C&S), 18.0 mm SL.

**Diademichthyinae – *Diademichthys***: *D.lineatus* – ROM 65282, 1 (C&S), 34.7 mm SL; ROM 74261, 1 (CT, https://doi.org/10.17602/M2/M30748), 35.0 mm SL; USNM 213595, 3 (C&S), 21.2–42.4mm SL. ***Lepadichthys***: *L.bolini* – ROM 55185, 2 (1 C&S; 1 CT, https://doi.org/10.17602/M2/M30731), 20.0–21.5 mm SL. *L.coccinotaenia* – SAIAB 49396, 3 (C&S), 28.4–39.1 mm SL; USNM 272920, 1 (C&S), 31.4 mm SL. *L.frenatus* – AMS I.27134-018, 1 (C&S), 28.0 mm SL. *L.lineatus* – ROM 72940, 2 (C&S), 29.1–36.5; SAIAB 9319, 2 (2 C&S), 23.5–25.0 mm SL.

**Diplocrepinae – *Aspasmogaster***: *A.costata* – AMS I.19103–015, 1 (C&S), 37.0 mm SL; TCWC 17166.01, 1 (CT, https://doi.org/10.17602/M2/M30754), 30.0 mm SL. *A.tasmaniensis* – ANSP 113616, 1 (C&S), 35.2 mm SL. ***Cochleoceps***: *C.orientalis* – AMS I.41084–007, 1 (C&S), 27.3 mm SL. *C.spatula* – WAM P.28288–002, 3 (C&S), 40.0–43.0 mm SL. *C.viridis* – WAM P.30262–001, 2 (C&S), 24.0–25.0 mm SL. ***Gastrocyathus***: *G.gracilis* – ANSP 113604, 1 (C&S), 32.5 mm SL; NMNZ P.035573, 2 (C&S), 21.0–23.0 mm SL. ***Gastrocymba***: *G.quadriradiata* – AMS I.21498-001, 1 (C&S), 27.5 mm SL; NMNZ P035573, 2 (C&S), – mm SL. ***Gastroscypthus***: *G.hectoris* – TCWC 17177.04, 2 (C&S), 30.0–31.0 mm SL. ***Parvicrepis***: *P.parvipinnis* – TCWC 17169.01, 4 (C&S), 16.0–19.0 mm SL. ***Pherallodus***: *P.indicus* – NSMT-P 114703, 1(CT; https://doi.org/10.17602/M2/M56337), 25.0 mm SL.

## Supplementary Material

XML Treatment for Flexor

XML Treatment for Flexor incus
